# Predicting pharmaceutical inkjet printing outcomes using machine learning

**DOI:** 10.1016/j.ijpx.2023.100181

**Published:** 2023-04-17

**Authors:** Paola Carou-Senra, Jun Jie Ong, Brais Muñiz Castro, Iria Seoane-Viaño, Lucía Rodríguez-Pombo, Pedro Cabalar, Carmen Alvarez-Lorenzo, Abdul W. Basit, Gilberto Pérez, Alvaro Goyanes

**Affiliations:** a Departamento de Farmacología, Farmacia y Tecnología Farmacéutica, I+D Farma (GI-1645), Facultad de Farmacia, Instituto de Materiales (iMATUS) and Health Research Institute of Santiago de Compostela (IDIS), Universidade de Santiago de Compostela, 15782, Spain; b Department of Pharmaceutics, UCL School of Pharmacy, University College London, 29-39 Brunswick Square, London WC1N 1AX, UK; c IRLab, CITIC Research Center, Department of Computer Science, University of A Coruña, Spain; d IRLab, Department of Computer Science, University of A Coruña, Spain; e FabRx Ltd., Henwood House, Henwood, Ashford TN24 8DH, UK; f Fabrx Artificial Intelligence, Carretera de Escairón, 14, Currelos (O Saviñao) CP 27543, Spain

**Keywords:** Additive manufacturing and personalized medications, 2D and 3D printed drug products, Artificial intelligence and digital health, Desktop ink jet printing of pharmaceuticals and drug delivery systems, Design and fabrication of medicinal products, Rational formulation development

## Abstract

Inkjet printing has been extensively explored in recent years to produce personalised medicines due to its low cost and versatility. Pharmaceutical applications have ranged from orodispersible films to complex polydrug implants. However, the multi-factorial nature of the inkjet printing process makes formulation (e.g., composition, surface tension, and viscosity) and printing parameter optimization (e.g., nozzle diameter, peak voltage, and drop spacing) an empirical and time-consuming endeavour. Instead, given the wealth of publicly available data on pharmaceutical inkjet printing, there is potential for a predictive model for inkjet printing outcomes to be developed. In this study, machine learning (ML) models (random forest, multilayer perceptron, and support vector machine) to predict printability and drug dose were developed using a dataset of 687 formulations, consolidated from in-house and literature-mined data on inkjet-printed formulations. The optimized ML models predicted the printability of formulations with an accuracy of 97.22%, and predicted the quality of the prints with an accuracy of 97.14%. This study demonstrates that ML models can feasibly provide predictive insights to inkjet printing outcomes prior to formulation preparation, affording resource- and time-savings.

## Graphical abstract

**Figure:**
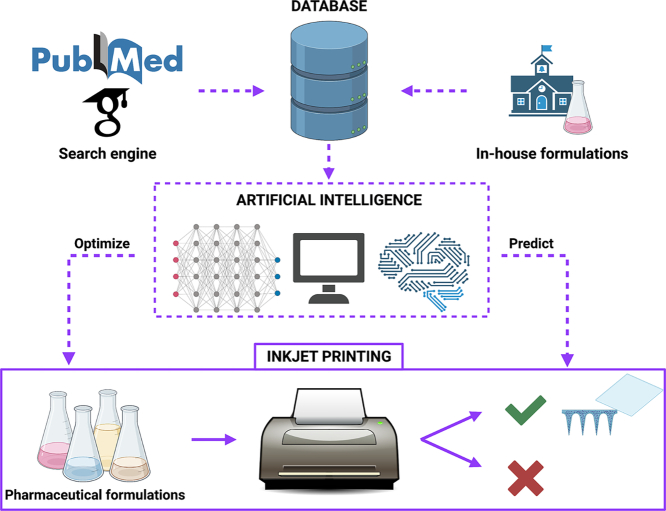

## 1 Introduction

Inkjet printing is a manufacturing technology based on material jetting, wherein droplets of ink are deposited onto a substrate. Inkjet printing has garnered considerable attention amongst pharmaceutical scientist for its versatility in producing personalised medicines and unique dosage forms (Alomari et al., 2015; Scoutaris et al., 2016a). Notably, inkjet printing has been used to load drugs onto orodispersible films (Alomari et al., 2018; Arshad et al., 2020; Jachowicz, 2017; Kiefer et al., 2021; Vuddanda et al., 2018), bioadhesive films for cervical administration (Varan et al., 2017), transdermal microneedles (Boehm et al., 2014; Uddin et al., 2015), coronary metal stents (Scoutaris et al., 2016b), contact lenses (Pollard et al., 2023; Tetyczka et al., 2022), and even nails (Pollard et al., 2022). Inkjet printing has also been used to dispense drug-loaded micro- and nanoparticles dispersed in the ink liquid (Akagi et al., 2014; Boehm et al., 2013; Chou et al., 2021; Lee et al., 2012; Yeo et al., 2004). Inkjet printing may also be combined with other additive manufacturing technologies to impart special features that would otherwise be unattainable with conventional manufacturing technologies. For instance, inkjet printing was used in conjunction with fused deposition modelling (FDM™) 3D printing to produce drug-loaded tablets with quick response (QR) codes printed on them (Trenfield et al., 2019). These QR codes were designed to encode patient-related information that could be read using a smartphone, and to serve as an anti-counterfeit strategy. A similar concept was also applied for the fabrication of orodispersible substrates and capsules with printed QR codes (Chao et al., 2021; Edinger et al., 2018; You et al., 2016). Potent drugs or drugs with low dose requirements are conventionally explored for inkjet printing due to the technology's suitability for printing low dose drug products. However, inkjet printing has also been used to fabricate the entire 3D drug-loaded tablets (Acosta-Vélez et al., 2018; Kyobula et al., 2017; Sen et al., 2020) and implants (Ruiz-Cantu et al., 2021). The affordability, precise control of droplet deposition, and versatility of inkjet printing has supported the continued expansion of research in its pharmaceutical applications, resulting in a wealth of publicly available data on printing parameters and outcomes (Evans et al., 2021).

In inkjet printing, droplets are deposited either continuously (continuous inkjet) or with a drop-on-demand (DoD) mechanism. DoD inkjet printing can be further categorised based on the mechanism by which droplets are generated: thermal and piezoelectric inkjet printing (Daly et al., 2015; Hoath, 2016; Singh et al., 2010). In thermal inkjet printing, a thermal resistor present in the printhead heats the ink, inducing rapid vaporisation and consequently the formation of a vapour bubble that forces a droplet out of the nozzle. In piezoelectric inkjet printing, an electric current is applied to a piezoelectric material in the printhead, inducing mechanical deformation of the material which in turn exerts a pressure within the printhead, thereby ejecting a droplet. Due to the absence of heat application, piezoelectric inkjet printing has been popularly explored for the fabrication of personalised medicines loaded with thermally labile drugs (Eleftheriadis et al., 2020a; Eleftheriadis et al., 2020b). Interestingly, while it might be instinctive to assume that thermal inkjet printing is not amenable to heat-sensitive drugs, there is insufficient evidence demonstrating that thermal degradation of drug occurs during the printing process. This is because the application of heat in the thermal inkjet printing process last for only a few milliseconds (Scoutaris et al., 2016a).

Regardless of the deposition mechanism, the optimization of ink characteristics and consequent printing outcomes have been the focus of pharmaceutical inkjet printing research (Evans et al., 2021). Ink properties, such as the viscosity, density, and surface tension, and printing parameters, such as printing speed and nozzle diameter, influence printing outcomes to different arbitrary degrees (Azizi Machekposhti et al., 2020). Undesirable printing results may include clogging, tailing of droplets, and the production of satellite droplets (tiny droplets splattered around the main droplet) (Azizi Machekposhti et al., 2019). Due to the plethora of variables, ink formulation development is often an empirical process that is time-, material-, and cost-consuming. Conventionally, the Ohnesorge number, which is a dimensionless number that describes the tendency of a droplet to stay intact, is used to predict if the ink will be jettable. An Ohnesorge number ranging between 0.1 and 1, which is equivalent to a Z value (where Z is the reciprocal of the Ohnesorge number) of 1–10, is often considered to be printable (Derby, 2015). However, there have been numerous exceptions, with inks with Z values above 10 found to be printable (Liu and Derby, 2019). Therefore, a predictive tool to better determine the printability of inks prior to the actual preparation and testing would allow pharmaceutical researchers to redirect their time and focus to devising more unique dosage forms to solve unmet clinical challenges.

Machine learning (ML) is a branch of artificial intelligence (AI) that studies how to provide machines with learning capacity, based on algorithms capable of identifying and learning from patterns in large and complex datasets. ML is one of the key enabling technologies of Industry 4.0, and has already transformed numerous industries by providing actionable insights that previous approaches strategies fail to provide (Rai et al., 2021). The additive manufacturing and personalised medicines community within the pharmaceutical sector has also begun exploring ways in which ML may be used to re-invent traditionally time-consuming processes (Elbadawi et al., 2021a; Elbadawi et al., 2021b; Jing et al., 2018). For example, ML has been used to predict printing outcomes and dissolution behaviours of FDM™-printed dosage forms (Elbadawi et al., 2020; Muñiz Castro et al., 2021; Obeid et al., 2021; Ong et al., 2022) and digital light processing (DLP)-printed tablets (Tagami et al., 2021), and to predict the design and fabrication of microneedle arrays (Rezapour Sarabi et al., 2022). However, ML-enabled predictive tools for inkjet printing outcomes have yet to be developed despite the pool of publicly available data in the literature.

Therefore, the present study aims to develop and evaluate the performance of ML models, using data mined from published literature, for predicting inkjet printing printability and the total drug dose in the final printed dosage form. This study will evaluate the multifactorial dependence of inkjet printing outcomes, and how ML may be used to analyse nuance differences and provide more reliable predictions as opposed to the conventional guidance on jettability based on Z values.

## 2 Materials & methods

### 2.1 Data collection

Google Scholar, PubMed, and Web of Science were used to extract articles published in English using the terms “inkjet printing” or “ink jet printing” or “ink-jet printing”, and “drug” or “drug device”, published between May 2000 and February 2022. For articles to be included in the dataset, they must meet the following criteria:

• Articles must include information about the composition of the ink formulation.
• Articles must include information that will allow the Z value to be calculated or information about the printer/nozzle/nozzle diameter.
• Articles must be reporting about drug printing, e.g. articles on cell printing were excluded.
• Articles reporting binder jetting were excluded.

Additionally, information of several in-house formulations was also included. The data collected can be divided into four groups: (1) *formulation composition and properties, (2) individual material properties*, (3) *process-related parameters*, and (4) *target variables*. These are further elaborated on in the following subsections.

#### 2.1.1 Formulation composition and properties

The composition of the excipients and drug in each formulation (ratio of the weight of each component to the total weight) were recorded. Attention was given in ensuring that for every formulation, the cumulative total ratio surmised to 1. Information on the physical properties of each formulation were also recorded in the dataset. These data are described in Table 1.

**Table 1:** Summary of formulation-related properties.

Variable	Description
Viscosity (mPa·s)	Resistance of the ink formulation to deformation at a given rate.
Surface tension (mN/m)	Energy required to remove the surface layer of ink in a unit area.
Density (g/mL)	Measure of the ink's mass per unit of volume.
Ohnesorge (Oh) number	Dimensionless value that describes the tendency for a droplet to stay intact. It represents the ratio of internal viscosity forces to the surface tension and inertial energy. The lower the Oh number, the more likely a droplet will be formed, vice versa.
Z value	Inverse of the Ohnesorge number.

#### 2.1.2 Individual material properties

The physicochemical properties of individual components of each formulation were also collected through *PubChem* and the *Handbook of Pharmaceutical Excipients (9th ed.)* (Sheskey et al., 2020). These were namely the material's molecular weight, melting point, and boiling point. If the material is a drug, its solubility in water was also recorded. Each component was also labelled with their material type, which are groups based on the materials' chemical structure. For example, the enteric polymers Eudragit E-100 and Eudragit RLPO are labelled with as “acrylic” material type, while rasagiline mesylate and terbinafine hydrochloride are labelled as “amine”. There was a total of 84 material types.

#### 2.1.3 Process-related parameters

Information on the parameters associated with the inkjet printing process were included in the dataset. These variables are described in Table 2.

**Table 2:** Summary of process-related variables.

Variable	Description
Object printed	Type of device that was being printed (e.g. film, particles, microneedles, tablets)
Printer	Model of inkjet printer that was used.
Nozzle	Model of nozzle that was used.
Nozzle Diameter	Size of the orifice through which droplets were ejected from.
Print frequency	Vibration frequency of the piezoelectric material in a piezoelectric inkjet printer.
Peak voltage	Voltage applied to the piezoelectric material in the piezoelectric printer.
Drop spacing (μm)	Distance between each droplet.
Reported droplet volume (pL)	Volume of an individual droplet ejected by the inkjet printer.
Area (cm^2^)	Surface size of the desired print.
Number of layers printed in a single print cycle	Total number of layers that was printed during one printing process.
Theoretical drug dose (mg)	The amount of drug expected to be contained in a single printed dosage form.

#### 2.1.4 Target variables

Target variables are the variables that the ML models are built to predict. In this study, these were whether the ink formulation was printable, if the ink were printable whether they would produce satellite droplets, and the total drug dose in the final printed dosage form (Table 3).

**Table 3:** Summary of target variables.

Target variables	Values	Analysis Type
Printability	Yes or No	Binary Classification
Printability (satellite)	Good or Satellite	Binary Classification
Total drug dose	Drug dose (mg)	Regression

### 2.2 Feature set generation

Based on the formulation composition (as described in Section 2.1.1) and/or individual material properties (as described in Section 2.1.2), five feature sets were generated: material with company name, material name, material type, weighted physical properties, and physical properties by material type. These feature sets differ in how information about the formulation composition is represented. These were created as previously reported, except for the weighted physical properties and physical properties by material type feature set (Ong et al., 2022). Briefly, in the material with company name feature set, both the tradename of the material and the company from which it is supplied are treated as unique identifiers (e.g., PLGA from Birmingham Polymers is regarded as different from PLGA from KITECH). On the other hand, the material name feature set regards the same material from different companies as the same. Using the same example, PLGA from Birmingham Polymers and that from KITECH will both be regarded as “PLGA”. The material type feature set also groups materials, but by their chemical structure rather than their name, thereby further reducing dimensionality. The weighted physical properties feature set was generated by calculating the weighted molecular weight, melting point, boiling point, and water solubility of the drugs. The values of the individual weighted properties were calculated by multiplying the physical property by the weight fraction of the material. For example, if 2.0% w/w of caffeine (MW = 194.19 g/mol) was used in the formulation, the weighted molecular weight of caffeine in this formulation would be 3.8838 g/mol (0.02 * 194.19). When a given physical property of a material is unknown, only the weight fraction and properties of the remaining materials that make up the formulation are used to compute the weighted average of that physical property. The physical properties by material type feature set was created by combining weighted physical properties and material type, where the input is the weighted physical properties for each material type in the formulation.

### 2.3 Machine Learning Techniques (MLTs)

A computer running a macOS Monterey operating system (v12.5), with an Apple M1 Max chip and installed RAM memory of 32GB, was used for data analysis and development of ML models described herein. All scripts reported herein were developed using python (v3.9.7) with the scikit-learn package (scikit-learn, v0.24.2).

#### 2.3.1 Data pre-processing

Any formulation with missing data was removed. To improve machine learning performance, quantile transformation was applied to numerical variables for them to have Gaussian distribution. Categorical variables were label encoded.

#### 2.3.2 Selecting best set of MLT, feature set, and additional input parameters

To develop a suitable machine learning model for each target variable, the best combination of MLT, feature set (as reported in Section 2.2), and additional input parameters (e.g., process-related parameters and formulation-related properties) was investigated. Three MLTs were used in this study: *artificial neural networks* (ANN)*, support vector machines* (SVM)*,* and *random forests* (RF). Every possible permutation of MLT, feature set, and relevant additional input parameters were evaluated for each target variable over 50 random seed values. A 75:25 split was used for training and testing the MLTs. Permutations that resulted in less than a third of the total number of formulations (i.e., 229 formulations) were excluded from evaluation. The default hyperparameter values for each MLT is described in Table 4.

**Table 4:** Default hyperparameter values used for each MLT in the initial ML experiment.

MLT	Hyperparameter	Value
Random Forests	Bootstrap	False
	Maximum depth	40
	Maximum features	Sqrt (square root)
	Minimum samples leaf	1
	Minimum samples split	2
	Number of estimators	10
Support Vector Machines	C (regularization parameter)	100
	Gamma (kernel coefficient)	0.1
	Kernel	Rbf (radial basis function)
Artificial Neural Networks	Hidden Layer Sizes	60, 40, 10
	Learning rate	Constant
	Solver	Adam (stochastic gradient-based optimizer)
	Activation	Relu (rectified linear unit function)
	Alpha	0.0001
	Maximum iterations	500

The performance of machine learning models developed from each permutation was evaluated based on numerous metrics depending on the type of analyses being conducted. For classification analyses, the Cohen's kappa, precision, recall, and F1 was used. For regression analysis, the mean absolute error (MAE) and the coefficient of determination (R^2^) was used. For each set of MLT, feature set, and additional input parameters, the average metric score over 50 seeds were calculated. The set that performed the best for each target variable was subsequently optimized by hyperparameter tuning.

#### 2.3.3 Hyperparameter tuning

A fixed set of possible values for each hyperparameter for each MLT was pre-defined (see respective tables in following sections). The best hyperparameter values for each MLT were determined from a grid search with 5-fold cross-validation, performed on the training set. The optimized machine learning models were then applied to the testing dataset, and their performance were evaluated based on the metrics described in Section 2.3.2.

## 3 Results

### 3.1 Exploratory data analysis

Using the search terms as described in Section 2.1, a total of 357 articles were found, although only 21.0% of these articles met the inclusion criteria. Consequently, a total of 687 formulations were extracted from 75 articles (full list enumerated in Supplementary Material Table S1) and 2 in-house projects. Reporting of information relevant to printing settings (e.g., print frequency, droplet volume) and formulation characteristics (e.g., viscosity, density) were very heterogenous. To improve the completeness of the dataset, the values of some parameters, namely dynamic or absolute viscosity (labelled in this study as viscosity), density, *Z*-value, and Ohnesorge number, were estimated if they were not reported. The density of the formulation was estimated by dividing the total mass of the materials used over the total volume of the ink formulation. Dynamic viscosity could be estimated in cases where the kinematic viscosity is reported by multiplying the kinematic viscosity (in mm^2^/s) with the density of the formulation (in g/mL). As articles typically only reported either the Z-value or the Ohnesorge number, and the Ohnesorge number is the reciprocal of the Z-value, one was used to calculate the other. Since the Z-value and the Ohnesorge number share the same information, with an ideal ML algorithm, there would be no difference between using one or the other, and using both should not add any improvement as they are redundant. However, because they are not on the same scale, either might be more suitable for a given algorithm, so both values were evaluated.

**Fig. 1:**
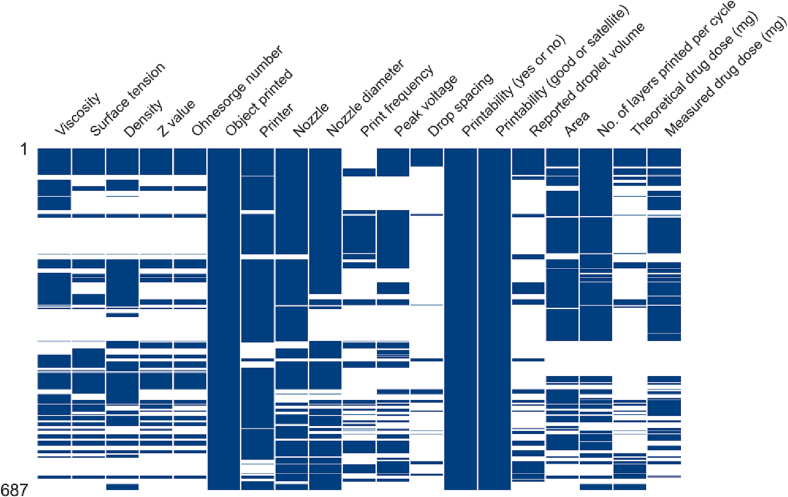
Missing matrix diagram, after populating dataset with estimated values. Blue bars indicate the availability of data, and missing data are indicated by white spaces. (For interpretation of the references to colour in this figure legend, the reader is referred to the web version of this article.)

Despite efforts to populate the dataset, availability of material- and process-related parameters remained diverse, as illustrated in Fig. 1. As formulations with missing data must be removed, the inclusion of all parameters for machine learning analysis is not possible as the resulting dataset will be too small. To identify the optimal balance between the number of parameters included as inputs and the preservation of as many formulations as possible, the residual data size of all possible combinations of material- and process-parameters were evaluated. Any combination that resulted in a loss >66% of the original number of formulations was disregarded.

**Fig. 2:**
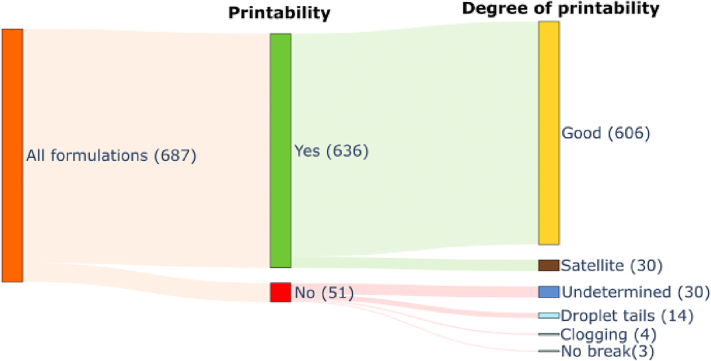
Sankey diagram showing distribution of printability.

Out of 687 formulations, 636 were printable (92.6% of all formulations), of which 30 produced satellite droplets (4.72% of printable formulations) (Fig. 2). This positively skewed dataset demonstrates the tendency for researchers to publish only positive results, as highlighted in our previous studies (Elbadawi et al., 2020; Muñiz Castro et al., 2021; Ong et al., 2022). This imbalance could be worsened when formulations with missing information are omitted. Therefore, when evaluating the best combination of MLT, feature set, material-, and process-related parameters, the ratio of positive to negative outcomes must also be considered.

**Fig. 3:**
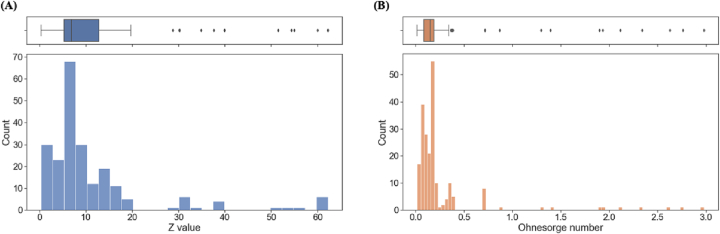
Histogram and boxplot of (A) *Z*-value of printable formulations, and (B) Ohnesorge number of printable formulations.

Amongst printable ink formulations, analysis of Z values and Ohnesorge numbers demonstrated exceptions to the conventional guidance that inks with 1 < Z < 10 were printable (Fig. 3). While printable formulations clustered within this range, there were 68 formulations with Z > 10, with the upper limit of 62.2 demonstrating significant deviation from the rule. Rather than supporting the guidance on Z value, this clustering may instead indicate a routine omission of formulations with Z values >10 from printing, resulting in an under-reporting of printable formulations with Z > 10. This supports the need for a better tool for predicting the printability of formulations for inkjet printing.

**Fig. 4:**
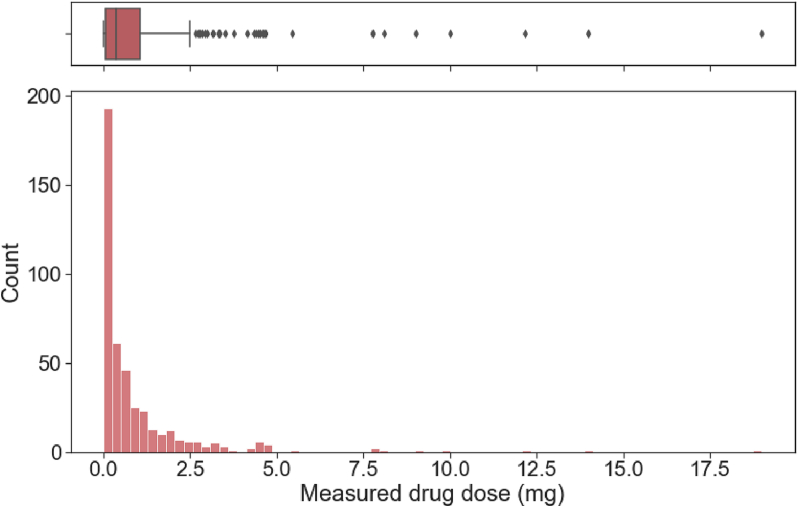
Histogram and boxplot of measured drug dose in printed formulations.

The amount of drug loaded in printed formulations ranged from 1.7 × 10^−4^ to 19 mg, with most formulations loaded with <2 mg of active pharmaceutical ingredient (Fig. 4). Apart from the concentration of drug in the ink formulation, variations in the measured drug dose also arise from differences in the size of the printed area, and the number of layers printed. While formulations with higher drug loadings might appear as anomalies, they should be retained in the training set since these formulations were intentionally loaded with larger amount of drug. Instead, the right-skewed distribution of drug loading demonstrates the need for quantile transformation to normalise the values for better machine learning performance.

**Fig. 5:**
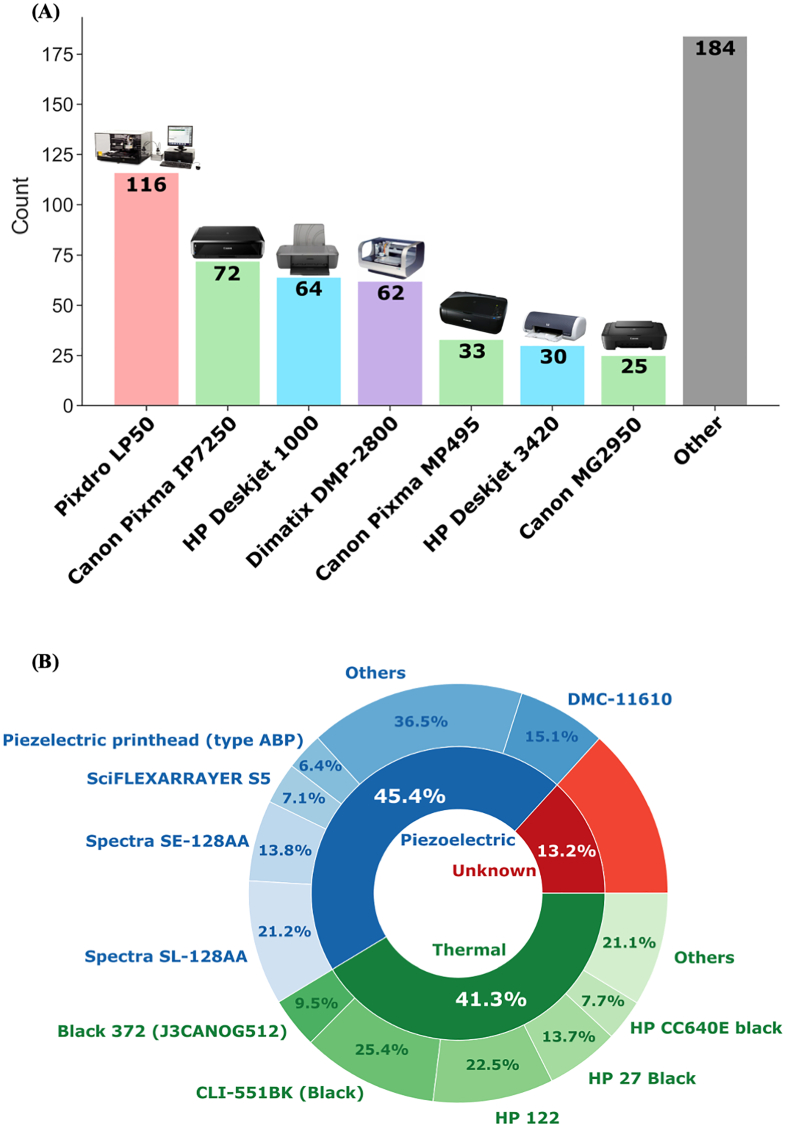
(A) Bar chart showing the seven most frequently used printers, arranged in rank order from left to right. Colours correspond to the printer brands, and printers not in the top seven were grouped as “others”. (B) Nested pie-chart showing the distribution of piezoelectric, thermal, and unknown printing technology used to fabricate the recorded formulations. The top five nozzles used for piezoelectric and thermal inkjet printing are reflected in the outer pie-chart.

The choice of inkjet printer invariably influences the printability of a given formulation as it may impose limitations on the printing parameters, such as the mechanism of printing and in some cases the nozzles compatible with the printer. Therefore, the models of inkjet printers that were used to fabricate these formulations were also explored. The seven most used inkjet printers are shown in Fig. 5A. However, it should be noted that 101 formulations did not report the inkjet printer that was used. Since the most used printer (Pixdro LP50) was used for 116 formulations, the reporting of these 101 unknown printers could influence this ranking and its absence could mask the popularity of less frequently used printers. Piezoelectric inkjet printing was also found to be marginally more popular than thermal inkjet printing, accounting for 45.4% and 41.3% of the collected formulations, respectively (Fig. 5B).

**Fig. 6:**
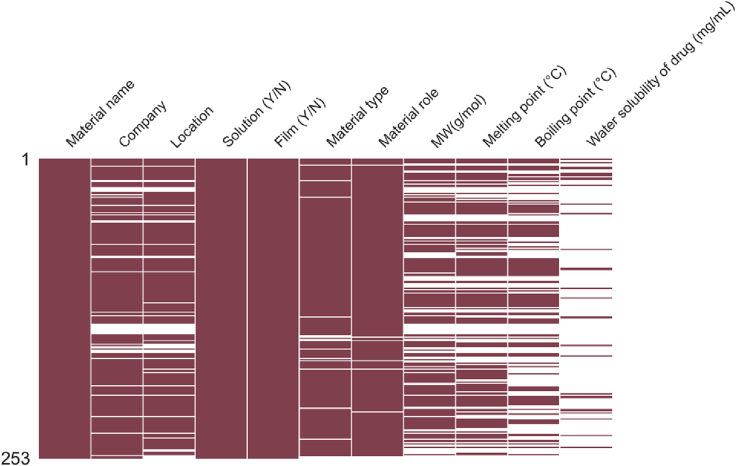
Missing matrix of the information on materials used in the extracted studies. Red bars indicate the availability of data, and missing data are indicated by white spaces. (For interpretation of the references to colour in this figure legend, the reader is referred to the web version of this article.)

Quantifiable characteristics of the materials used in each formulation was crucial for generating the *weighted physical properties* and *physical properties by material type* feature sets. In this study, the melting point, boiling point, molecular weight, and water solubility (for active pharmaceutical ingredients only) for each material was extracted from publicly accessible databases such as PubChem and published literature. As illustrated in Fig. 6, the availability of these data for the 253 materials used in the extracted studies was heterogenous. Materials with missing information on molecular weight, melting point, and boiling point were largely proprietary materials, such as commercial inks and Soluplus, where such information were not made publicly available by the respective companies. While Fig. 6 might suggest sparse availability of information on water solubility of drugs, this is largely because most materials used were excipients.

**Fig. 7:**
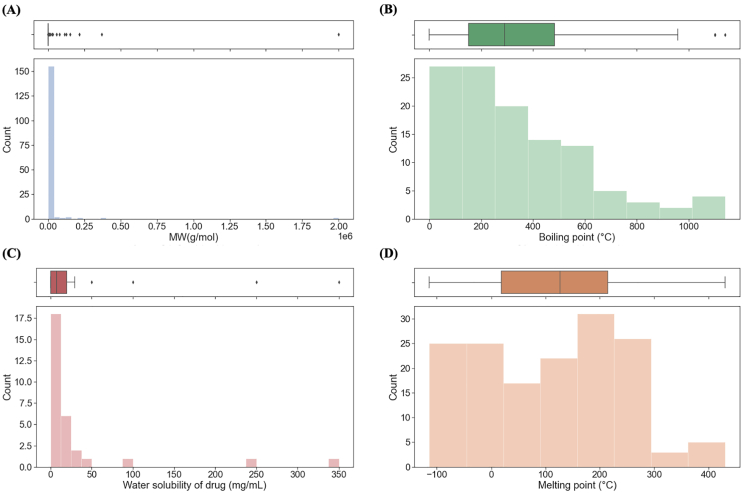
Histogram and boxplot of (A) molecular weight, (B) boiling point of all materials, (C) water solubility of active pharmaceutical ingredients, and (D) melting point of all materials.

The molecular weights of materials ranged from 1.8 × 10^1^ to 2 × 10^6^ g/mol, and has a right-skewed distribution with 54.9% of materials (139 out of 253) possessing molecular weights <1000 g/mol (Fig. 7A). Materials with larger molecular weights (>10^5^ g/mol) were biomolecules (proteins and polysaccharides) such as hydroxypropylcellulose and ribonuclease-A, and polymers such as poly(DL-lactic acid) (PDLA) and poly(lactic acid) (PLLA). The material with the highest molecular weight (2 × 10^6^ g/mol) was Gantrez™ AN-169, which is a polymer used as a film base material. As materials with large molecular weights were not anomalies, quantile transformation was again applied to normalise the values for better machine learning performance. Right-skewed distribution was also observed for boiling point and water solubility values, which ranged from 5.66 × 10^−3^ to 1140.4 °C and 1 × 10^−3^ to 350 mg/mL, respectively (Fig. 7B & C). On the other hand, distribution of melting point values appears bimodal, ranging from −114.1 to 430.5 °C (Fig. 7D). To prevent MLTs from assigning greater weights to values that are significantly numerically larger, normalization by quantile transformation was also applied to all numerical variables.

### 3.2 Machine learning model development & evaluation

#### 3.2.1 Printability (yes or no)

**Fig. 8:**
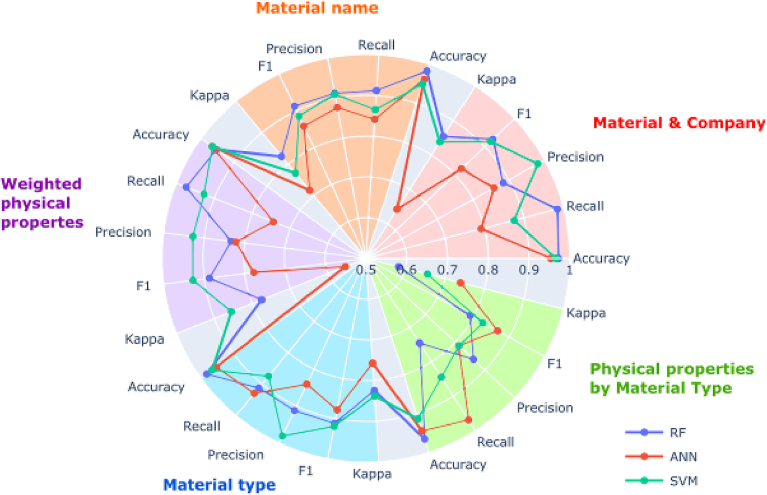
Radar plot with metrics results of models predicting printability (yes or no).

Initial model evaluation over 50 seeds using pre-defined hyperparameters found that the best algorithm for binary prediction of formulation printability was *random forest* (Fig. 8). The best performance was attained using the *grouped by material and company* feature set, coupled with information on *object printed*, *printer*, and *number of printed layers per cycle*. This configuration resulted in a residual dataset size of 486 formulations (70.7% of all formulations), 2.88% of which were negative outcomes (not printable). Machine learning performance is significantly influenced by the balance of targeted variables in the dataset, where an imbalanced dataset is expected to yield less reliable performance. Given the lower proportion of negative outcomes compared to the original dataset (7.42%), this configuration along with any that resulted in <7.42% negative printability outcomes were disregarded. With this criterion, the best performance was again attained using *random forest* and the *grouped by material and company* feature set, but now coupled with information on *nozzle diameter*, *nozzle*, and *printer*. This configuration gave a dataset comprising 429 formulations (62.4% of all formulations), of which 9.32% were negative outcomes. Therefore, the machine learning model was trained on a better-balanced dataset and is expected to produce more reliable predictions than a model trained with the original dataset. A suitable predictive tool should be capable of forecasting printability prior to any formulation preparation, as the time- and resource-savings afforded would otherwise be insignificant. Therefore, it is important that any input parameters must be quantifiable or determinable a priori. In the best performing model for predicting printability, all additional parameters fit this criterion.

This model was subsequently optimized by tuning the hyperparameters, with the fixed set of possible values and best values for each hyperparameter summarised in Table 5. The optimized model had an *accuracy* of 97.22% and a *Cohen's kappa coefficient* of 0.854. As the *Cohen's kappa coefficient* accounts for the possibility of making a correct prediction by chance, the score obtained by the optimized model indicates high predictive reliability despite the relatively unbalanced training dataset.

**Table 5:** Optimal hyperparameters for random forest model for predicting printability (yes or no).

Hyperparameter	Possible values	Best value
Bootstrap	True, False	False
Criterion	Gini, Entropy	Entropy
Max depth	7, 15, 40, None	40
Max features	Auto, Sqrt	Sqrt
Minimum samples leaf	1, 2, 4	1
Minimum samples split	2, 5, 10	2
No. of estimators	5, 10, 20, 30, 60, 100	20

#### 3.2.2 Printability (good or satellite)

**Fig. 9:**
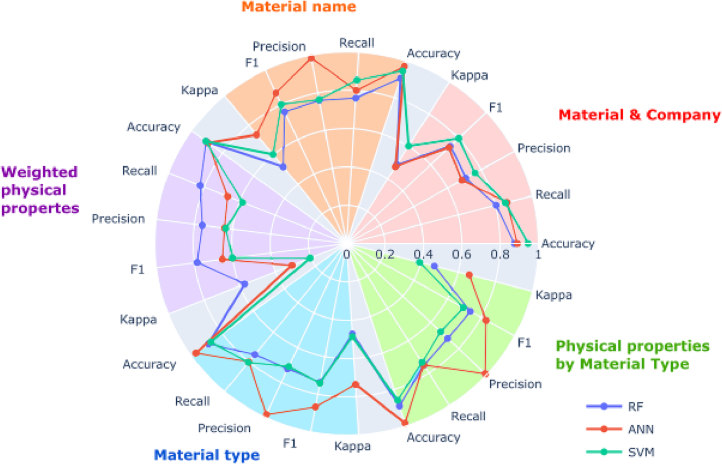
Radar plot with metrics results of models predicting printability (good or satellite).

Printable formulations, while jettable, may produce satellite droplets that are undesirable as they lead to messy and imprecise printing. Therefore, after predicting if the formulations were printable, it was important for our machine learning pipeline to predict if the jettable formulations produced satellite droplets. For training and evaluating these models, non-printable formulations were excluded entirely. This gave an original dataset comprising 636 formulations, of which 3.80% were satellite outcomes. Multilayer perceptron (ANN) was found to be the best MLT based on initial model evaluation over 50 seeds using pre-defined hyperparameters (Fig. 9). The best performance was attained using the *grouped by material type* feature set, coupled with information on *nozzle diameter* and *printer*. This configuration resulted in a residual dataset size of 412 formulations (64.8% of printable formulations), 1.70% of which were negative outcomes (satellite droplets).

As in Section 3.2.1., since this configuration gave a lower proportion of negative outcomes (satellite droplets) compared to the original dataset (4.72%), it was disregarded. Instead, amongst configurations that gave at least the same proportion of negative outcomes as the original dataset, the best performance was again attained using multilayer perceptron (ANN) and the *grouped by material type* feature set. However, this was now coupled with information on *density*, *object printed*, and *nozzle*. This configuration gave a dataset comprising 280 formulations (44.3% of all formulations), of which 7.50% were negative outcomes. All parameters used in this configuration can be determined a priori, with density being estimable as described in Section 3.1. The optimized ANN model achieved an *accuracy* of 97.14%, and a *Cohen's kappa coefficient* of 0.74, using optimized hyperparameter values summarised in Table 6.

**Table 6:** Optimal hyperparameters for multilayer perceptron model for predicting printability (good or satellite).

Hyperparameter	Possible values	Best value
Activation	Relu, Identity, Logistic, Tanh	Relu
Alpha	0.0001, 0.001, 0.005	0.001
Hidden layer sizes	(100, 50, 10), (60, 40, 10), (80, 30, 10)	60, 40, 10
Learning rate	Constant, Inverse scaling, adaptive	Constant
Maximum iterations	500	500
Solver	Adam, Limited-memory Broyden-Fletcher-Goldfarb-Shanno algorithm (LBFGS), Stochastic Gradient Drescent	LBFGS

#### 3.2.3 Measured drug dose

Initial model evaluation over 50 seeds using pre-defined hyperparameters found that the best algorithm for prediction of the measured drug dose in the printed product was *random forest*, using the *grouped by material name* feature set. The additional material- and process-related parameters considered were the *object printed, printer, area,* and *number of printed layers per cycle*. This configuration resulted in a residual dataset size of 405 formulations (59.0% of all formulations, and 93.1% of formulations with reported measured drug dose). Notably, upon removing the minimum threshold required for the residual dataset size, the best algorithm was ANN, using the *grouped by material name* feature set with a residual dataset size of 83 (19.1% of formulations with reported measured drug dose). The additional material- and process-related parameters were also different; they were the *surface tension, density, Z value, Ohnesorge number, printer, number of printed layers per cycle, and theoretical drug dose*. This model performed better than the initial *random forest* model using the larger dataset: R^2^ = 0.911 vs 0.769, and MAE = 0.184 vs 0.282. In both cases, *printer* and *number of printed layers per cycle* were fed into the algorithm, which agrees with conventional thinking as these two parameters directly influence the amount of material that is being deposited, and hence the drug dose loaded into the printed objects.

**Fig. 10:**
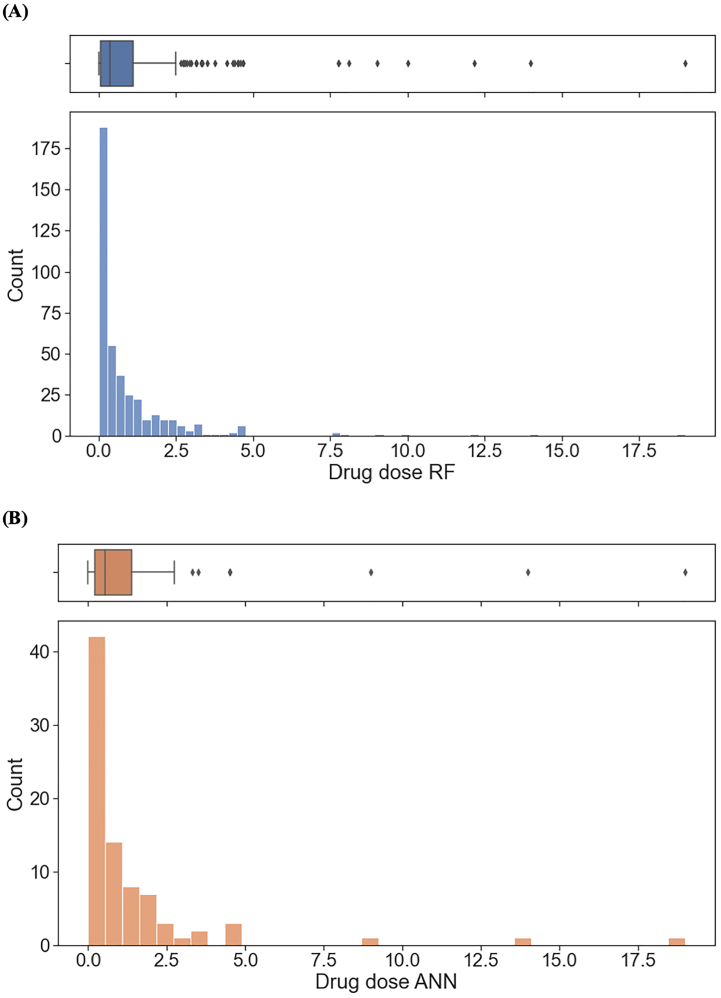
Histogram and boxplot of measured drug dose in dataset used to train (A) RF model and (B) ANN model.

Interestingly, the distribution of the measured drug doses of the two resulting residual datasets are similar (Fig. 10). However, from the box plots, the dataset used to train the RF model possessed a relatively higher proportion of data ranging between 2.5 and 5.0 mg compared to that used to train the ANN model. Drug doses in this range are commonly explored in pharmaceutical inkjet printing and are not outliers as implied by the histogram plots. As such, the dataset used to train the RF model has a higher proportion of data that falls within the typical range of drug doses used in pharmaceutical inkjet printing. Therefore, while the ANN model performed slightly better than the RF model, the latter was deemed more suitable for optimization as it was trained on a larger dataset and should therefore be more robust.

Hyperparameter tuning of this random forest-based model yielded values as summarised in Table 7. The optimized model had an R^2^ of 0.800 and a MAE of 0.291, indicating that it could provide predictions of the actual drug loading within ±0.291 mg. Considering that the mean drug load was 0.944 mg (and the median drug load was 0.36 mg), significant improvement to the model is necessary for practical deployment. However, this result is within expectations given the right-skewed distribution of drug doses. Performance could conceivably be improved with a more normally distributed dataset.

**Table 7:** Optimal hyperparameters for random forest model for predicting total measured drug dose.

Hyperparameter	Possible values	Best value
Bootstrap	True, False	False
Criterion	MSE, MAE	MAE
Max depth	7, 15, 40, None	50
Max features	Auto, Sqrt	Sqrt
Minimum samples leaf	1, 2, 4	1
Minimum samples split	2, 5, 10	2
No. of estimators	10 to 60 (inclusive)	19

## 4 Discussion

Formulation development and optimization is a time- and resource-intensive process that can be considerably accelerated by guidance from predictive in silico tools. The current guidance states that only inks with Z values <10 are printable. Following this guidance produces a false positive rate of 77.42%, a false negative rate of 28.16%, and an accuracy of 64.39%, based on the same dataset used for training and testing the ML model for predicting printability (excluding 224 formulations with no known Z values) (Table 8). In comparison, the optimized model for predicting printability performed significantly better, with an accuracy of 97.22%. This highlights the multifactorial dependence of inkjet printing outcomes, and the importance of considering numerous material- and process-related parameters beyond a formulation's Z value in making such predictions.

**Table 8:** Confusion matrix based on conventional guidance.

	Actual: Printable	Actual: Not printable
Predicted: Printable	125	24
Predicted: Not printable	49	7

ML models were also successfully developed that provided reliable predictions on whether satellite droplets will be formed. These predictions were not previously possible based on the conventional guidance. Interestingly, neither models for predicting printing outcomes nor satellite droplet formation included the formulations' Z value or Ohnesorge number as inputs, despite the current guidance for inkjet printing printability being based on these variables. Exploratory data analysis on the Z values of extracted formulations also demonstrated how some inks (31.05% of printable formulations with known Z values) remained jettable despite possessing a Z value >10. These findings support the assertion that inkjet printing outcomes cannot be determined solely based on a formulation's Z value or Ohnesorge number. The long-held guidance on Z values could have also resulted in an undertesting of formulations with Z > 10. Therefore, the ML models developed in this study could be further enhanced with more data on such formulations.

A recent study demonstrated that for piezoelectric inkjet printing, alterations to the pulse shape can influence the formation of satellite droplets (Zettl et al., 2023). Unfortunately, as the observation was only reported recently, pharmaceutical inkjet printing articles that are available in the public domain have not reported this parameter. The sparse reporting of pulse shape therefore precludes its inclusion in the present study. However, following the findings made and reported by Zettl et al., frequent reporting of the parameter's value in articles hereon could conceivably improve the performance of the ML model for predicting droplet quality developed in the present study.

Admittedly, the dataset consolidated in this study is considerably imbalanced, owing to the tendency for researchers to only publish positive results. A balanced dataset is critical for optimal machine learning performance, as it ensures that the model has sufficient training instances for all possible outcomes. As demonstrated in our previous study, even slight to moderate improvements to the balance of datasets can improve ML performance significantly (Ong et al., 2022). Therefore, the models developed in this study can be further enhanced with more negative data, which researchers are encouraged to report, either in the supplementary materials section of their article or privately amongst the community.

It is worth noting that the dataset consolidated in this study is more than two times smaller than that of our previous study that focused on predicting FDM™ 3D printing outcomes (Ong et al., 2022), 687 vs 1594 formulations respectively. This is despite inkjet printing being a more mature technology than FDM™ 3D printing, bearing testament to the underutilisation of inkjet printing as a manufacturing technology for personalised drug-loaded products. This is likely due to inkjet printing stereotypically being deemed to only be suitable for low dose but highly potent drugs. However, recent studies have proven its capability in printing entire objects, including tablets and complex implants, which will likely inspire more expansive research in pharmaceutical inkjet printing. With higher volume and better-balanced reporting of pharmaceutical 3D printing data, a machine learning model with good generalizability may be developed and deployed on a web server. Akin to the free web-based software that was created for predicting FDM™ printing outcomes (*M3DISEEN*), this will then accelerate inkjet printing formulation development and consequently inkjet printing research in general.

## 5 Conclusion

In this study, ML models were successfully developed for predicting printing outcomes of inkjet printing and the drug load of the printed objects. Analysis of the dataset comprising 687 formulations from literature-mined and in-house studies revealed that positive printing outcomes were overwhelmingly published in favour of negative outcomes. Despite the imbalanced dataset, the optimized ML model for predicting printability performed significantly better than the conventional guidance based on Z values. ML models for predicting satellite droplet formation also provided reliable predictions, offering predictive insights that were previously unattainable. To further enhance ML performance, the publishing or sharing of negative data is highly encouraged. In doing so, a highly reliable in silico tool, such as a web-based software, may be deployed to accelerate pharmaceutical inkjet printing research, allowing researchers to focus on novel inkjet printing solutions for urgent unmet clinical needs.

## Funding

The research was partially supported by MCIN (PID 2020-113881RB-I00/AEI/10.13039/501100011033), Spain, 10.13039/501100010801Xunta de Galicia (ED431C 2020/17), and FEDER.

## Declaration of Competing Interest

The authors declare the following financial interests/personal relationships which may be considered as potential competing interests:

Carmen Alvarez-Lorenzo reports financial support was provided by Spain Ministry of Science and Innovation. Carmen Alvarez-Lorenzo reports financial support was provided by Spanish Federation of Rare Diseases. Alvaro Goyanes reports a relationship with FabRx that includes: equity or stocks. Abdul Basit reports a relationship with FabRx that includes: equity or stocks. Co-author is an editor for International Journal of Pharmaceutics: X - A.W.B. Co-author is an editor for International Journal of Pharmaceutics: X - C.A.L.

## Data Availability

Data will be made available on request.

## Appendix A Supplementary data

List of published articles used in this study Image 1
