# Supplementary material for: Predicting pharmaceutical inkjet printing outcomes using machine learning
Source: Int J Pharm X. 2023 Apr 17;5:100181. doi: 10.1016/j.ijpx.2023.100181 (PMC10151423; doi:10.1016/j.ijpx.2023.100181)
Supplement: Supplementary file 1 — List of published articles used in this study [file mmc1.docx]

**Predicting Pharmaceutical Inkjet Printing Outcomes using Machine Learning**

Paola Carou-Senra^1,a^, Jun Jie Ong^2,a^, Brais Muñiz Castro^3^, Iria Seoane-Viaño^2^, Lucía Rodríguez-Pombo^1^, Pedro Cabalar^4^, Carmen Alvarez-Lorenzo^1^, Abdul W. Basit^2,5,*^, Gilberto Pérez^3,*^, Alvaro Goyanes^1,2,5,6,*^

^1^ Departamento de Farmacología, Farmacia y Tecnología Farmacéutica, I+D Farma (GI-1645), Facultad de Farmacia, Instituto de Materiales (iMATUS) and Health Research Institute of Santiago de Compostela (IDIS), Universidade de Santiago de Compostela, 15782, Spain.

^2^ Department of Pharmaceutics, UCL School of Pharmacy, University College London, 29-39 Brunswick Square, London WC1N 1AX, UK.

^3^ IRLab, CITIC Research Center, Department of Computer Science, University of A Coruña, Spain

^4^ IRLab, Department of Computer Science, University of A Coruña, Spain.

^5^ FabRx Ltd., Henwood House, Henwood, Ashford TN24 8DH, UK

^6^ Fabrx Artificial Intelligence, Carretera de Escairón, 14, Currelos (O Saviñao) CP 27543, Spain

^a^ These authors contributed equally to this work.

* Correspondence: a.basit@ucl.ac.uk (A.W.B.); a.goyanes@fabrx.co.uk (A.G.), gilberto.pvega@udc.es (G. P).

**Table S1. List of articles from which formulations were extracted**

| **No.** | **Authors** | **Title** | **DOI** |
| --- | --- | --- | --- |
| 1 | Genina et al. | Behavior of printable formulations of loperamide and caffeine on different substrates—Effect of print density in inkjet printing | 10.1016/j.ijpharm.2013.06.003 |
| 2 | Wickström et al. | Improvement of dissolution rate of indomethacin by inkjet printing | 10.1016/j.ejps.2015.03.009 |
| 3 | Pardeike et al. | Nanosuspensions as advanced printing ink for accurate dosing of poorly soluble drugs in personalized medicines. | 10.1016/j.ijpharm.2011.08.033 |
| 4 | Buanz et al. | Ink-jet printing versus solvent casting to prepare oral films: Effect on mechanical properties and physical stability | 10.1016/j.ijpharm.2014.12.032 |
| 5 | Buanz et al. | Preparation of Personalized-dose Salbutamol Sulphate Oral Films with Thermal Ink-Jet Printing | 10.1007/s11095-011-0450-5 |
| 6 | Vuddanda et al. | Personalisation of warfarin therapy using thermal ink-jet printing | 10.1016/j.ejps.2018.02.002 |
| 7 | Meléndez et al. | Thermal inkjet application in the preparation of oral dosage forms: Dispensing of prednisolone solutions and polymorphic characterization by solid-state spectroscopic techniques | 10.1002/jps.21189 |
| 8 | Genina et al. | Evaluation of different substrates for inkjet printing of rasagiline mesylate | 10.1016/j.ejpb.2013.03.017 |
| 9 | Wimmer-Teubenbacher  et al. | Pharmaceutical-grade oral films as substrates for printed medicine | 10.1016/j.ijpharm.2018.05.041 |
| 10 | Raijada et al. | A step toward development of printable dosage forms for poorly soluble drugs | 10.1002/jps.23678 |
| 11 | Planchette et al. | Printing medicines as orodispersible dosage forms: Effect of substrate on the printed micro-structure | 10.1016/j.ijpharm.2015.10.054 |
| 12 | Vakili et al. | Application of a colorimetric technique in quality control for printed pediatric orodispersible drug delivery systems containing propranolol hydrochloride | 10.1016/j.ijpharm.2016.07.032 |
| 13 | Kyobula et al. | 3D inkjet printing of tablets exploiting bespoke complex geometries for controlled and tuneable drug release | 10.1016/j.jconrel.2017.06.025 |
| 14 | Lee et al. | Fabrication of drug-loaded polymer microparticles with arbitrary geometries using a piezoelectric inkjet printing system | 10.1016/j.ijpharm.2012.02.011 |
| 15 | Akagi et al. | Inkjet printing of layer-by-layer assembled poly (lactide) stereocomplex with encapsulated proteins | 10.1021/la404162h |
| 16 | Kiefer et al. | Fundamental Investigations into Metoprolol Tartrate Deposition on Orodispersible Films by Inkjet Printing for Individualised Drug Dosing | 10.3390/_pharmaceutics13020247 |
| 17 | Alomari et al. | Printing T3 and T4 oral drug combinations as a novel strategy for hypothyroidism | 10.1016/j.ijpharm.2018.07.062 |
| 18 | Eleftheriadis et al. | Development and characterization of inkjet printed edible films for buccal delivery of B-complex vitamins | 10.3390/ph13090203 |
| 19 | Öblom et al. | Data-enriched edible pharmaceuticals (DEEP) of medical cannabis by inkjet printing | 10.1016/j.ijpharm.2020.119866 |
| 20 | Vakili et al. | Application of a handheld NIR spectrometer in prediction of drug content in inkjet printed orodispersible formulations containing prednisolone and levothyroxine | 10.1016/j.ijpharm.2017.04.014 |
| 21 | Turković et al. | An investigation into mechanical properties and printability of potential substrates for inkjet printing of orodispersible films | 10.3390/pharmaceutics13040468 |
| 22 | Edinger et al. | Visualization and Non-Destructive Quantification of Inkjet-Printed Pharmaceuticals on Different Substrates Using Raman Spectroscopy and Raman Chemical Imaging | 10.1007/s11095-017-2126-2 |
| 23 | Boehm et al. | Inkjet printing of amphotericin B onto biodegradable microneedles using piezoelectric inkjet printing | 10.1007/s11837-013-0574-7 |
| 24 | Eleftheriadis et al. | Fabrication of Mucoadhesive Buccal Films for Local Administration of Ketoprofen and Lidocaine Hydrochloride by Combining Fused Deposition Modeling and Inkjet Printing | 10.1016/j.xphs.2020.05.022 |
| 25 | Palo et al. | Development of oromucosal dosage forms by combining electrospinning and inkjet printing | 10.1021/acs.molpharmaceut.6b01054 |
| 26 | Pollard et al. | Printing drugs onto nails for effective treatment of onychomycosis | 10.3390/pharmaceutics14020448 |
| 27 | Montenegro-Nicolini et al. | Inkjet printing of proteins: an experimental approach | 10.1208/s12248-016-9997-8 |
| 28 | Acosta-Vélez et al. | Photocurable bioink for the inkjet 3D pharming of hydrophilic drugs | 10.3390/bioengineering4010011 |
| 29 | Yeo et al. | A new process for making reservoir-type microcapsules using ink-jet technology and interfacial phase separation | 10.1016/j.jconrel.2003.08.021 |
| 30 | Fakhfouri et al. | Drop-On-Demand Inkjet Printing of SU-8 Polymer | 10.2174/1876402910901010063 |
| 31 | Hoath et al. | How PEDOT: PSS solutions produce satellite-free inkjets | 10.1016/j.orgel.2012.10.004 |
| 32 | Gu et al. | Inkjet printed antibiotic-and calcium-eluting bioresorbable nanocomposite micropatterns for orthopedic implants | 10.1016/j.actbio.2011.08.006 |
| 33 | Dong et al. | An experimental study of drop-on-demand drop formation | 10.1063/1.2217929 |
| 34 | Sandler et al. | Inkjet printing of drug substances and use of porous substrates‐towards individualized dosing | 10.1002/jps.22526 |
| 35 | Boehm et al. | Inkjet printing for pharmaceutical applications | 10.1016/j.mattod.2014.04.027 |
| 36 | Eleftheriadis et al. | Inkjet printing of a thermolabile model drug onto FDM-printed substrates: formulation and evaluation | 10.1080/03639045.2020.1788062 |
| 37 | Cader et al. | Water-based 3D inkjet printing of an oral pharmaceutical dosage form | 10.1016/j.ijpharm.2019.04.026 |
| 38 | Tetyczka et al. | Itraconazole nanocrystals on hydrogel contact lenses via inkjet printing: implications for ophthalmic drug delivery | 10.1021-acsanm.2c01715 |
| 39 | Junqueira et al. | Coupling of fused deposition modeling and inkjet printing to produce drug loaded 3D printed tablets | 10.3390/pharmaceutics14010159 |
| 40 | Edinger et al. | QR encoded smart oral dosage forms by inkjet printing | 10.1016/j.ijpharm.2017.11.052 |
| 41 | Arshad et al. | Preparation and characterization of indomethacin loaded films by piezoelectric inkjet printing: a personalized medication approach | 10.1080/10837450.2019.1684520 |
| 42 | Clark et al. | Making tablets for delivery of poorly soluble drugs using photoinitiated 3D inkjet printing | 10.1016/j.ijpharm.2019.118805 |
| 43 | Genina et al. | Tailoring controlled-release oral dosage forms by combining inkjet and flexographic printing techniques | 10.1016/j.ejps.2012.07.020 |
| 44 | Vakili et al. | Hyperspectral imaging in quality control of inkjet printed personalised dosage forms | 10.1016/j.ijpharm.2014.12.034 |
| 45 | Clark et al. | 3D printing of tablets using inkjet with UV photoinitiation | 10.1016/j.ijpharm.2017.06.085 |
| 46 | Scoutaris et al. | Development and biological evaluation of inkjet printed drug coatings on intravascular stent | 10.1021/acs.molpharmaceut.5b00570 |
| 47 | Seera et al. | Evaluation of Cocrystallization Outcomes of Multicomponent Adducts: Rapid Fabrication to Achieve Uniform Particle Size Distribution Using Thermal Inkjet Printing | 10.1021/acs.cgd.0c00469 |
| 48 | Öblom et al. | Towards Printed Pediatric Medicines in Hospital Pharmacies: Comparison of 2D and 3D-Printed Orodispersible Warfarin Films with Conventional Oral Powders in Unit Dose Sachets | 10.3390/pharmaceutics11070334 |
| 49 | Trenfield et al. | Track-and-trace: Novel anti-counterfeit measures for 3D printed personalized drug products using smart material inks | 10.1016/j.ijpharm.2019.06.034 |
| 50 | Slabov et al. | Controlled Growth of Stable β-Glycine via Inkjet Printing | 10.1021/acs.cgd.9b00308 |
| 51 | Lopez-Iglesias et al. | From the printer to the lungs: Inkjet-printed aerogel particles for pulmonary delivery | 10.1016/j.cej.2018.09.159 |
| 52 | Fox et al. | Picoliter‐volume inkjet printing into planar microdevice reservoirs for low‐waste, high‐capacity drug loading | 10.1002/btm2.10053 |
| 53 | Thabet et al. | Continuous inkjet printing of enalapril maleate onto orodispersible film formulations | 10.1016/j.ijpharm.2018.04.064 |
| 54 | Boehm et al. | Inkjet deposition of itraconazole onto poly (glycolic acid) microneedle arrays | 10.1116/1.4941448 |
| 55 | Uddin et al. | 3D printed microneedles for anticancer therapy of skin tumours | 10.1016/j.msec.2019.110248 |
| 56 | Boehm et al. | Polyglycolic acid microneedles modified with inkjet-deposited antifungal coatings | 10.1116/1.4913378 |
| 57 | Edinger et al. | Quantification of inkjet-printed pharmaceuticals on porous substrates using Raman spectroscopy and near-infrared spectroscopy | 10.1208/s12249-019-1423-y |
| 58 | Huanbutta et al. | Manufacture of 2D-printed precision drug-loaded orodispersible film prepared from tamarind seed gum substrate | 10.3390/app11135852 |
| 59 | Kiefer et al. | Comparative investigations on key factors and print head designs for pharmaceutical inkjet printing | 10.1016/j.ijpharm.2020.119561 |
| 60 | Iftimi et al. | Edible solid foams as porous substrates for inkjet-printable pharmaceuticals | 10.1016/j.ejpb.2019.01.004 |
| 61 | Mau et al. | Adjusting inkjet printhead parameters to deposit drugs into micro-sized reservoirs | 10.1515/cdbme-2016-0086 |
| 62 | Economidou et al. | 3D printed microneedle patches using stereolithography (SLA) for intradermal insulin delivery | 10.1016/j.msec.2019.04.063 |
| 63 | Pissinato-Pere  et al. | 3D printed microneedles for insulin skin delivery | 10.1016/j.ijpharm.2018.03.031 |
| 64 | Eleftheriadis et al. | In vitro evaluation of 2D-printed edible films for the buccal delivery of diclofenac sodium | 10.3390/ma11050864 |
| 65 | Lion et al. | Customisable tablet printing: The development of multimaterial hot melt inkjet 3D printing to produce complex and personalised dosage forms | 10.3390/pharmaceutics13101679 |
| 66 | Nemeth et al. | Bottom-up fabrication of multilayer enteric devices for the oral delivery of peptides | 10.1007/s11095-019-2618-3 |
| 67 | Stranzinger et al. | Near-infrared hyperspectral imaging as a monitoring tool for on-demand manufacturing of inkjet-printed formulations | 10.1208/s12249-021-02091-x |
| 68 | Chen et al. | Multifunctional surfaces with biomimetic nanofibres and drug-eluting micro-patterns for infection control and bone tissue formation | 10.22203/ecm.v024a17 |
| 69 | Taresco et al. | Rapid Nanogram Scale Screening Method of Microarrays to Evaluate Drug–Polymer Blends Using High-Throughput Printing Technology | 10.1021/acs.molpharmaceut.7b00182 |
| 70 | Rosqvist et al. | A low-cost paper-based platform for fast and reliable screening of cellular interactions with materials | 10.1039/c9tb01958h |
| 71 | Algahtani et al. | Establishing a New Method to Evaluate the Recrystallization of Nanogram Quantities of Paracetamol Printed as a Microarray Using Inkjet Printing | 10.1021/acs.cgd.8b01121 |
| 72 | Varan et al. | Inkjet printing of antiviral PCL nanoparticles and anticancer cyclodextrin inclusion complexes on bioadhesive film for cervical administration | 10.1016/j.ijpharm.2017.04.036 |
| 73 | Zakharyuta et al. | Inkjet printing platform for fabrication of uniform, excipient-free drug particles for pulmonary delivery in a preclinical setting | 10.1016/j.jddst.2019.101226 |
| 74 | Choi et al. | Multicomponent high-throughput drug screening via inkjet printing to verify the effect of immunosuppressive drugs on immune T Lymphocytes | 10.1038/s41598-017-06690-2 |
| 75 | Machekposhti et al. | Patterned surfaces with the controllable drug doses using inkjet printing | 10.1557/s43578-021-00135-3 |
